# Protonated Form: The Potent Form of Potassium-Competitive Acid Blockers

**DOI:** 10.1371/journal.pone.0097688

**Published:** 2014-05-20

**Authors:** Hua-Jun Luo, Wei-Qiao Deng, Kun Zou

**Affiliations:** 1 Hubei Key Laboratory of Natural Products Research and Development, College of Chemistry & Life Science, China Three Gorges University, Yichang, Hubei, China; 2 Dalian Institute of Chemical Physics, Chinese Academy of Sciences, Dalian, Liaoning, China; Wake Forest University, United States of America

## Abstract

Potassium-competitive acid blockers (P-CABs) are highly safe and active drugs targeting H^+^,K^+^-ATPase to cure acid-related gastric diseases. In this study, we for the first time investigate the interaction mechanism between the protonated form of P-CABs and human H^+^,K^+^-ATPase using homology modeling, molecular docking, molecular dynamics and binding free energy calculation methods. The results explain why P-CABs have higher activities with higher pKa values or at lower pH. With positive charge, the protonated forms of P-CABs have more competitive advantage to block potassium ion into luminal channel and to bind with H^+^,K^+^-ATPase via electrostatic interactions. The binding affinity of the protonated form is more favorable than that of the neutral P-CABs. In particular, Asp139 should be a very important binding site for the protonated form of P-CABs through hydrogen bonds and electrostatic interactions. These findings could promote the rational design of novel P-CABs.

## Introduction

The gastric H^+^,K^+^-ATPase (proton pump), primarily responsible for gastric acid secretion, is the key therapeutic target for the ulcer diseases such as duodenal ulcers, gastric ulcers, gastro esophageal reflux disease (GERD), Zollinger-Ellison syndrome (Z-E), and gastritis [1]–[3]. As a member of the P_2_-type ATPase family, H^+^,K^+^-ATPase is a dimeric heterodimer composed of *α* subunit of about 1035 amino acids with 10 transmembrane (TM) segments and *β*-subunit glycoprotein with 290 amino acids [4], [5]. By cyclic phosphorylation and dephosphorylation of the catalytic subunit, H^+^,K^+^-ATPase undergoes conformational changes between E_1_ and E_2_. With phosphorylation, H^+^,K^+^-ATPase E_1_ conformation binding hydronium ion E_1_P•H_3_O^+^ changes to E_2_P•H_3_O^+^ form. After release of H_3_O^+^ and binding of K^+^ on the extracytoplasmic surface, the E_2_P•K^+^ conformation is formed and then converts to the E_1_K conformation with the dephosphorylation. The E_1_K conformation releases K^+^ to the cytoplasmic side, then rebinding of H_3_O^+^ occurs to complete the transition cycle [6]. The H^+^, K^+^-ATPase engages in 2K^+^/2H^+^/1ATP electroneutral ion exchange, generating a million-fold H^+^-gradient across the mammalian canalicular membrane of the parietal cell [7], [8].

H^+^,K^+^-ATPase inhibitors include two classes: proton pump inhibitors (PPIs) and potassium-competitive acid blockers (P-CABs) [9], [10]. PPIs such as omeprazole, lansoprazole, rabeprazole, pantoprazole, tenatoprazole and leminoprazole, are irreversible inhibitors of H^+^,K^+^-ATPase, which form a covalent complex with the protein at specific cysteine residues [11], [12]. Although currently recognized as the most effective drugs for the treatment of acid-related diseases, PPIs exhibit a delayed onset of acute effect and achieve full effect only slowly and incrementally over several dose cycles [13]. Therefore, many patients with GERD symptom are unsatisfied with PPIs treatment [14], [15]. An alternative to PPIs is P-CABs, which reversibly inhibit gastric H^+^,K^+^-ATPase by competing with the K^+^ on the luminal surface [1]. After oral dosing, P-CABs rapidly achieve high plasma concentrations with a fast onset of action [13].

**Figure 1:**
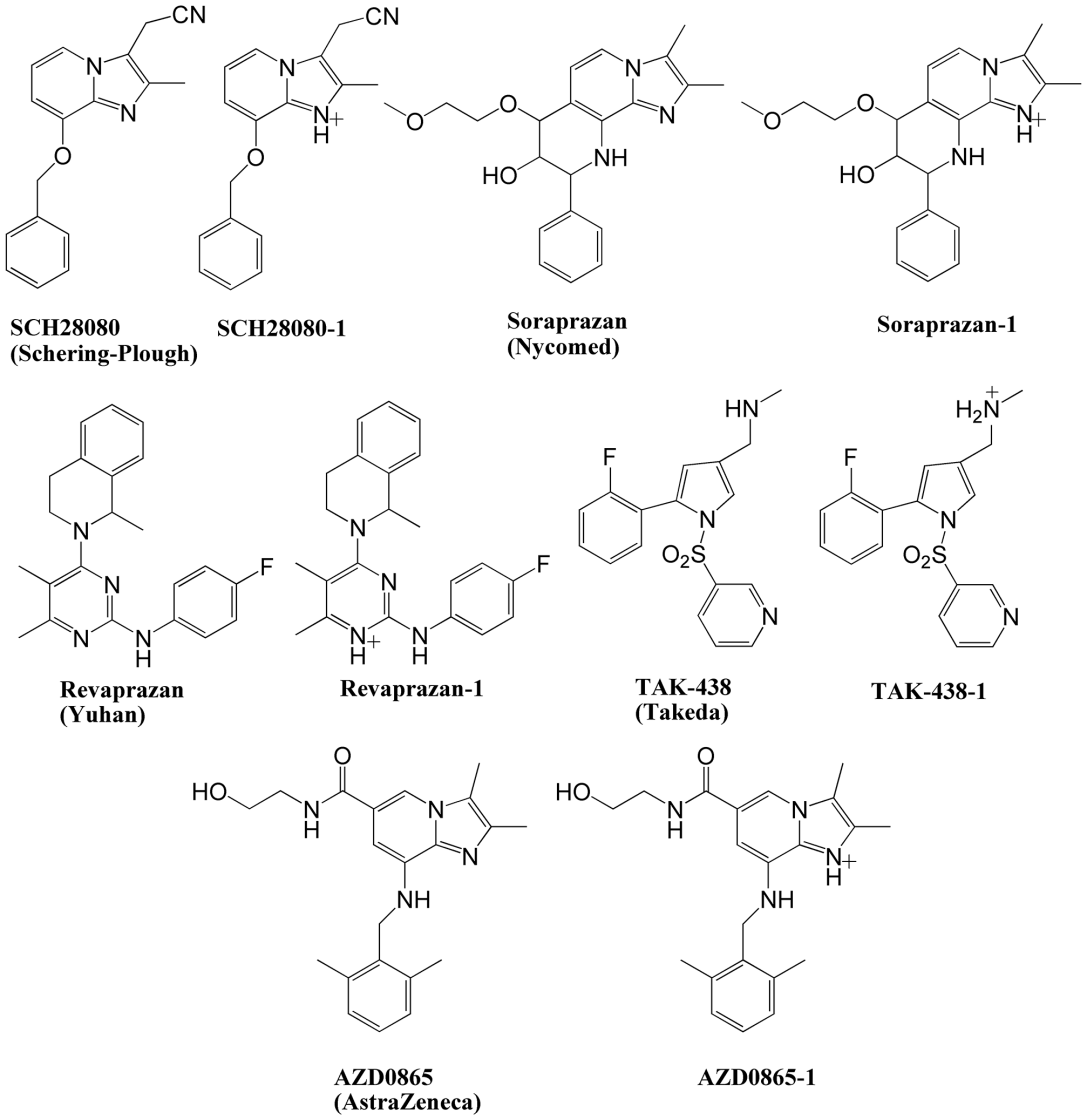
Chemical structures of P-CABs with their protonated form.

Now several P-CABs including SCH28080 [16]–[18], Soraprazan [19], [20], Revaprazan [21]–[23], AZD0865 [24], [25] and TAK-438 [26]–[29] (Figure 1) were developed by pharmaceutical companies. Among them Revaprazan was used clinically in 2007 for the treatment of duodenal ulcer, gastric ulcer and gastritis, and is undergoing phase III clinical studies for the treatment of GERD. All P-CABs are weak bases. SCH28080, AZD0865 and TAK-438 have pKa values of 5.6 [30], 6.1 [24] and 9.37 [27], respectively (Table 1). Gedda et al. [24] reported that at pH 7.4 AZD0865 concentration-dependently inhibited K^+^-stimulated H^+^,K^+^-ATPase activity (IC_50_ = 1.0 µM) but was more potent at pH 6.4 (IC_50_ = 0.13 µM). The theoretical protonated AZD0865 is approximately 33% at pH 6.4 and less than 5% at pH 7.4. SCH28080 has also been reported to be weaker under neutral conditions (IC_50_ = 0.14 µM at pH 6.5; IC_50_ = 2.5 µM at pH 7.5). In comparison, because of its high pKa value, TAK-438 should be protonated instantly and exert a potent inhibitory activity even in a neutral environment (IC_50_ = 0.019 µM at pH 6.5; IC_50_ = 0.028 µM at pH 7.5) [27]. According to the pKa calculation using ACD/I-Lab [31], SCH28080 and TAK-438 are 8.08% and 98.36% protonated at pH 6.5, while 0.87% and 94.67% protonated at pH 7.5 (Table 1). Thus, the protonated form would be the highly active form of P-CABs. But the mechanism of interaction between protonated P-CABs and H^+^,K^+^-ATPase is not known in detail, which hinders the development of novel P-CABs.

**Table 1:** IC_50_, pKa (reference) and pKa (calculation) values of P-CABs.

P-CABs	IC_50_/µM		pKa(Ref.)	pKa(Cal.)	protonated form (+1) percentage/%^a^	
	pH<7.0	pH≥7.0			pH 6.5	pH 7.5
SCH28080	0.14 (pH 6.5)	2.5 (pH 7.5)	5.6[ref.30]	5.85±0.10	8.08	0.87
Soraprazan		0.1 (pH 7.0)		7.11±0.70	95.50^b^	72.84
Revaprazan	0.35 (pH 6.1)			7.26±0.10	69.89^c^	10.42
AZD0865	0.13 (pH 6.4)	1.0 (pH 7.4)	6.1[ref.24]	6.50±0.10	33.00	5.00
TAK-438	0.019 (pH 6.5)	0.028 (pH 7.5)	9.37[ref.27]	9.06±0.10	98.36	94.67

a protonated form (+1) percentages were calculated using ACD/I-Lab except for AZD0865 which from reference [24]; b. protonated form percentage of Soraprazan is 87.07% at pH 7.0; c. protonated form percentage of revaprazan is 69.89% at pH 6.1.

So far the structural investigations of H^+^,K^+^-ATPase have lagged behind the pharmacological studies. The structure of human gastric H^+^,K^+^-ATPase is unknown, and the structure of pig gastric H^+^,K^+^-ATPase is poorly defined, being currently limited to a resolution of 7 Å (PDB code: 3IXZ [32], resolution: 6.5 Å; PDB code: 2XZB [33], resolution: 7 Å). So the aim of this study is to model H^+^,K^+^-ATPase structure by homology modeling and for the first time to investigate the interactions between the protonated form of P-CABs and H^+^,K^+^-ATPase using molecular docking, molecular dynamics and MM/GBSA calculation methods.

## Materials and Methods

### Homology modeling

The sequences of the human (1035 amino acids) and pig (1033 amino acids) gastric H^+^,K^+^-ATPase receptor were taken from the Swiss-Prot Database (ID: P20648 and P09626) [34]. Through BLAST online method (http:// blast.ncbi.nlm.nih.gov/Blast.cgi) [35] from the Protein Data Bank [36], the crystal structure of Na^+^,K^+^-ATPase in the E_2_P state (PDB code: 2ZXE) [37] was used as a template. The sequence alignment was performed with the ClustalW2 algorithm [38]. The homology model of human H^+^,K^+^-ATPase was generated using MODELLER9v4 [39]. The resultant structure of the H^+^,K^+^-ATPase was subject to the Protein Preparation Wizard module in Schrödinger [40] as follows: adding hydrogens, assigning partial charges, and minimizing using the OPLS-2005 force field [41] until RMSD 0.30 Å. The final optimized model was validated using the program PROCHECK [42] to assess the quality of the stereochemistry of the protein structure.

### Preparation of ligands

LigPrep of Schrödinger software suit [43] was used for the preparation of ligands: generating 3D structures from 2D (SDF) representation, and performing a geometry minimization. The ligands were subjected to energy minimization using MacroModel module of Schrödinger with Merck Molecular Force Field (MMFFs). Truncated Newton Conjugate Gradient (TNCG) minimization method was used with 500 iterations and convergence threshold of 0.05 (kJ/mol). While Epik [44] was used to generate possible ionization states at pH 7.0±1.0.

### Molecular docking

The docking simulations were performed using the software Glide (XP mode) [45], [46]. Previous biochemical and mutagenesis studies [18], [33], [47]–[50] suggest that Cys813 in pig H^+^,K^+^-ATPase (corresponding to Cys815 in human H^+^,K^+^-ATPase) is the key amino acid residue in the luminal cavity. Therefore, dimensions for the cubic boundary box centered on the centroid of Cys815 were set to 20 Å×20 Å×20 Å. The scaling factor for protein van der Waals radii was 1.0 in the receptor grid generation. After docking calculations, for each ligand, the best pose was chosen and scored using the proprietary GlideScore function.

### QM/MM optimization

The final docking complexes were energetically optimized by QM/MM method. QM/MM calculations were carried out using the QSite program [51], [52] of the Schrödinger suite. The ligands were defined as QM region calculated by the density functional theory DFT/B3LYP (6-31G* basis set). The receptor as MM region was minimized with Truncated Newton algorithm (maximum cycles as 1000; gradient criterion as 0.01). The OPLS 2005 all-atom force field was employed.

### Molecular dynamics

The optimized docking models were subjected to molecular dynamics simulations using Desmond [53], [54]. The system was embedded in a fully hydrated POPC (1-palmitoyl-2oleoyl-*sn*-glycero-3-phosphatidylchlorine) bilayer membrane and solvated with an orthorhombic box of SPC water molecules (buffer distance: 10 Å×10 Å×12 Å). Counter-ions (Na^+^) were added to neutralize the system and a physiological salt concentration of 0.15 M NaCl was introduced. The final system was composed of approximately 130,000 atoms. Each model was equilibrated in MD for 10 ns. Then two K^+^ were added in the luminal domain near channel (close to Asp134) replacing two Na^+^, and potassium ion competition molecular dynamics simulations were carried out for 10 ns. Before the simulation, the models were relaxed as follows: (1) two minimization steps (restraining the solute and unrestrained minimization) with maximum runs of 2000 and the convergence threshold for minimization set to 1 kcal/mol/Å. The minimization method was a hybrid of the steepest decent and limited-memory Broyden-Fletcher-Goldfarb-Shanno (LBFGS) algorithms; (2) after minimization, the simulation in the NVT ensemble was run restraining all solute heavy atoms with temperature of 10 K for 20 ps, using Berendsen thermostat; (3) a simulation in the NPT ensemble restraining all solute heavy atoms with temperature of 10 K and 300K for 50 ps, respectively; (4) a simulation in the NPT ensemble, no restraints, with temperature of 300 K and simulation time of 200 ps. Then 10 ns MD production runs (time step: 2.0 fs) were performed through NPT ensemble at 300 K with 1.0132 bar pressure. Smooth particle mesh Ewald method (Ewald tolerance: 1e-09) was employed to treat the long-range electrostatic interactions and a 9 Å radius cut off was used for coulombic short range interactions. The energies and frames of each trajectory were recorded every 1 ps and 5 ps, respectively. After 10 ns MD simulations with two K^+^, SCH28080 and TAK438-1 systems with H^+^,K^+^-ATPase and POPC membrane in 3M KCl resolution were equilibrated in MD for 40 ns and then run 100 ns disassociation molecular dynamics, respectively. MD trajectory analysis was performed using Desmond utilities and VMD [55]. The ligand-protein complexes were visualized using PyMOL [56] and analyzed with Ligand Interactions module embedded in Maestro 9.3 [57].

### MM/GBSA calculations

For each system, binding free energy (ΔG_bind_) calculations were performed for 100 snapshots extracted from the last 1 ns stable MD trajectory using molecular mechanics-generalized Born surface area (MM/GBSA) method. MM/GBSA procedure in Prime program [58], [59] was used to calculate ΔG_bind_ of the docked inhibitors according to the following equations [60]:
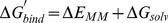
(1)

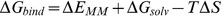
(2)Where ΔE_MM_ is the difference of the gas phase MM energy between the complex and the sum of the energies of the protein and inhibitor, and includes ΔE_internal_ (bond, angle, and dihedral energies), ΔE_Elect_ (electrostatic), and ΔE_VDW_ (van der Waals) energies. ΔG_solv_ is the change of the solvation free energy upon binding, and includes the electrostatic solvation free energy ΔG_GB_ (polar contribution calculated using generalized Born model), and the nonelectrostatic solvation component ΔG_SA_ (nonpolar contribution estimated by solvent accessible surface area). TΔS is the change of the conformational entropy upon binding. This term was calculated using normal-mode analysis Rigid Rotor Harmonic Oscillator (RRHO) contained in MacroModel module [61]. ΔG^'^_bind_ neglects the effect of entropy contributions, while ΔG_bind_ includes contributions from loss of ligand translational, rotational and vibrational entropy (TΔS).

## Results and Discussion

### H^+^,K^+^-ATPase homology model

**Figure 2:**
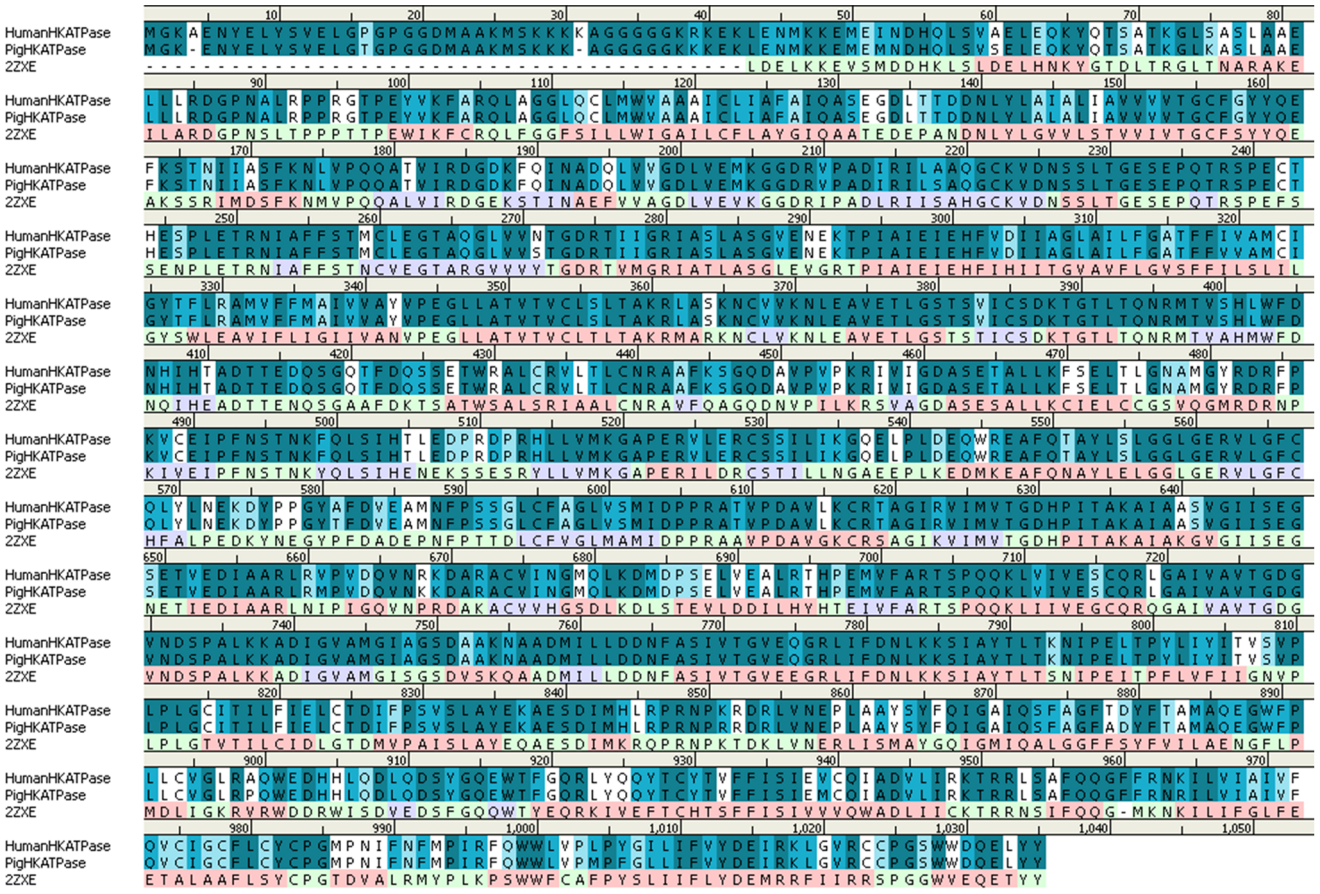
Sequence alignment results between human H^+^,K^+^-ATPase and the template Na^+^,K^+^-ATPase (2ZXE). The residues with identical, strong and weak similarities in H^+^,K^+^-ATPase are shown in dark blue, blue and light blue color background, respectively. The alpha helical, sheet and coil of secondary structure in the template are colored by pink, light purple and light green.

**Figure 3:**
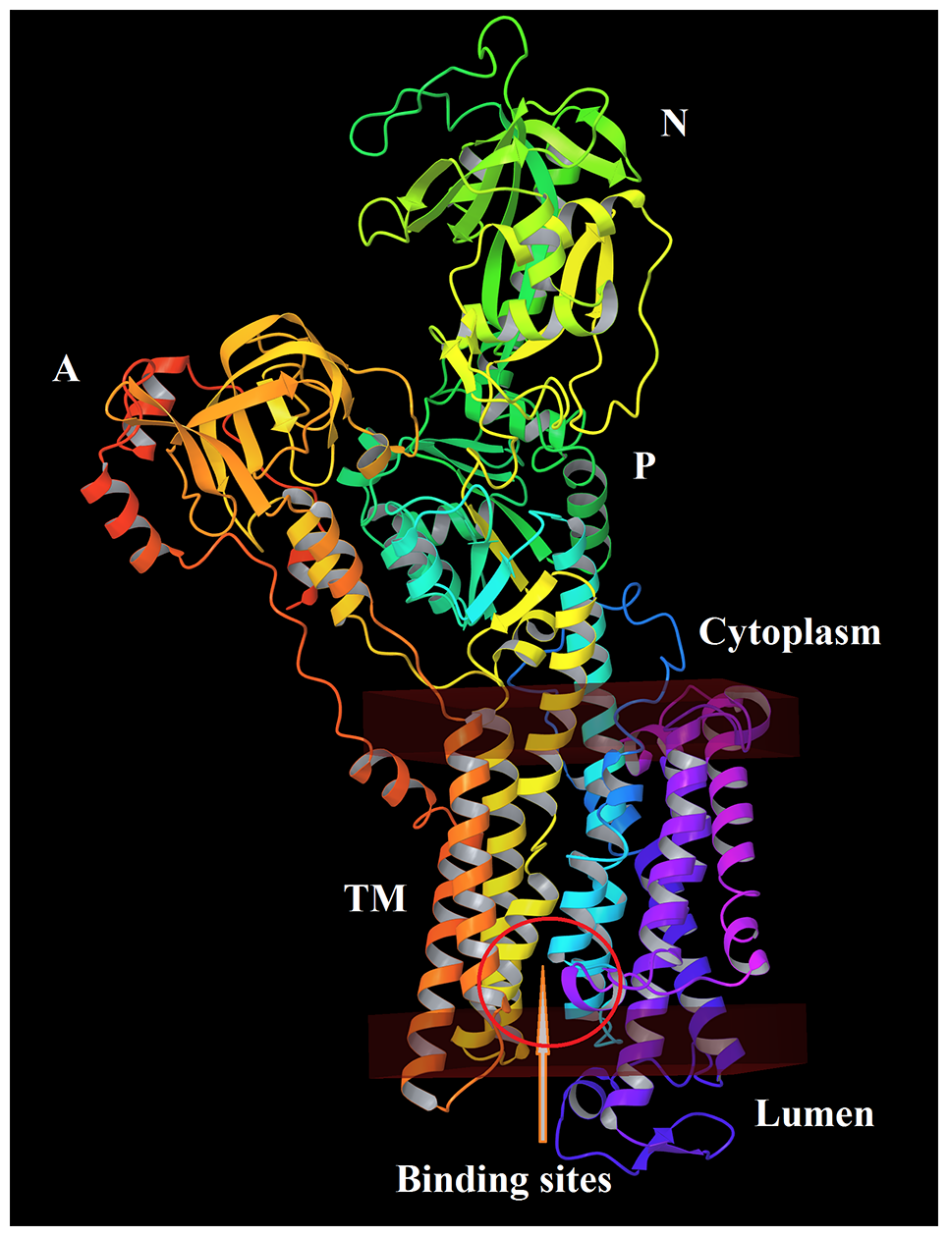
The human gastric H^+^,K^+^-ATPase model.

The three-dimensional structure of Na^+^,K^+^-ATPase in the E_2_P state (PDB code: 2ZXE; resolution: 2.4 Å) [37] was selected as a template, which share 63.9% identity and 78.7% similarity to human H^+^,K^+^-ATPase on the basis of sequence alignment analysis (Figure 2). The human gastric H^+^,K^+^-ATPase model is shown in Figure 3. It comprises ten transmembrane helices (TM1–TM10), in which binding sites are located, and three cytoplasmic domains: the nucleotide-binding (N), phosphorylation (P) and actuator (A) domains [33]. The stereochemistry of the homology model was assessed using Ramachandran plot, which indicates that 93.8% of the residues were located in the most favored zones, 6.1% in allowed regions, 0.0% in generously allowed regions and 0.1% in disallowed regions (Figure S1). The dihedrals, covalent and overall G-factors of this model are -0.17, 0.01 and -0.08, respectively. The PROCHECK G-factor should be above -0.5 ideally for the homology model to have a good acceptance score.

### Molecular dynamics simulations

**Figure 4:**
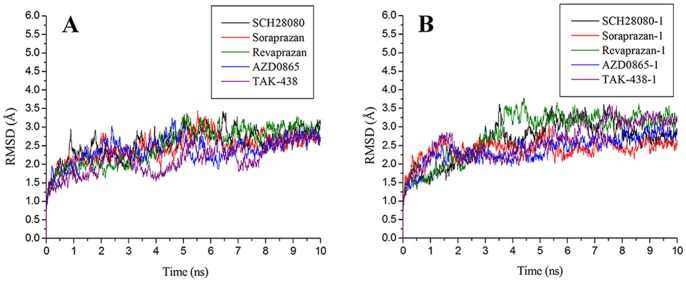
RMSDs for the backbone atoms of the complexes: (A) P-CABs; (B) protonated form of P-CABs.

**Figure 5:**
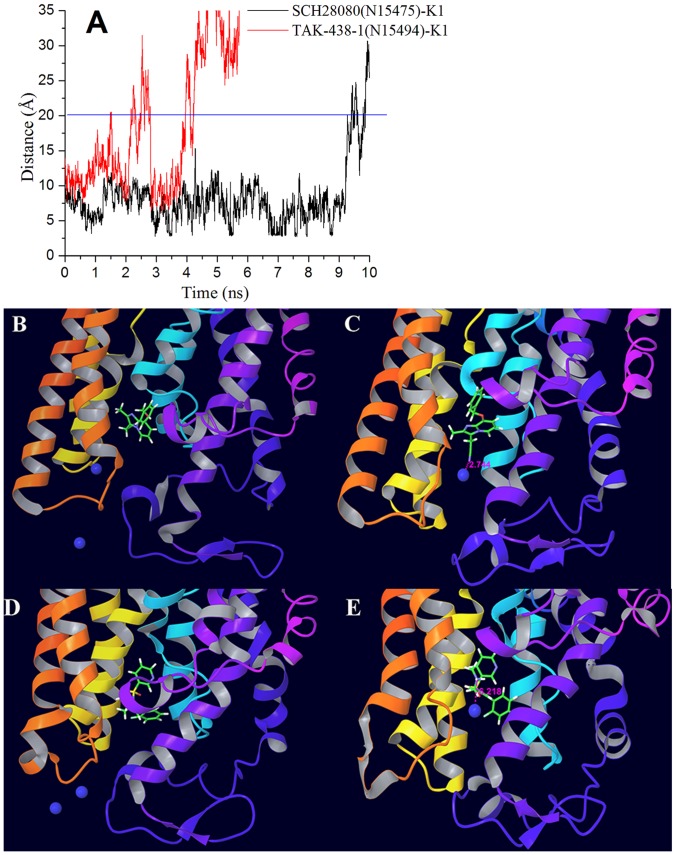
Time evolutions of the distances between potassium ion (K1) and nitrogen atom of P-CABs (N15475 of SCH28080 or N15494 of TAK-438-1) (A). The distances beyond 35 Å of TAK-438-1 are not shown in plot. The interaction modes of SCH28080 system at 0 ns (B) and 7.570 ns (C), and TAK-438-1 system at 0 ns (D) and 3.180 ns (E), with the potassium ion represented by blue ball.

**Figure 6:**
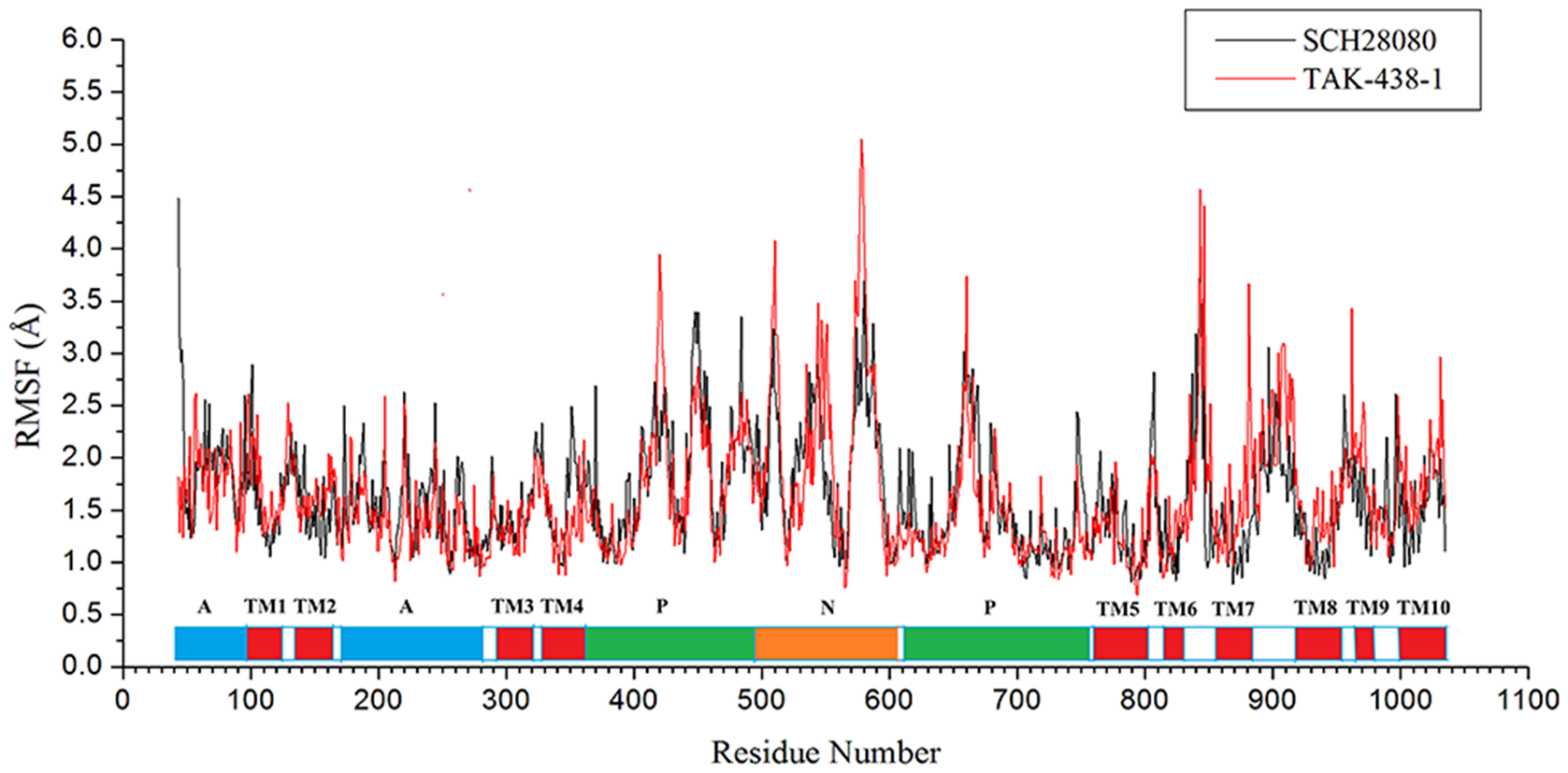
RMSF of each residue in SCH28080 and TAK-438-1 complexes.

After molecular docking and QM/MM optimization (Glide docking Gscore, Glide energy and QM/MM energy were listed in Table S1), MD simulations for ten P-CAB-H^+^,K^+^-ATPase complexes with two K^+^ in NaCl aqueous solution were run for a duration of 10 ns. To explore the dynamic stability of complexes and to ensure the rationality of the sampling method, root-mean-square deviations (RMSD) for the backbone atoms from the starting structure were analyzed, as shown in Figure 4. As can be seen in the plots, after 8 ns, the RMSD of each system tends to converge, indicating that the systems are stable and equilibrated. During the whole MD simulation process, the potassium ions were blocked into the luminal channel by all P-CABs, which acted just like goalkeepers. The distances between one of potassium ions (K1) and the nitrogen atom (N15475) of –CN in SCH28080 (or nitrogen atom (N15494) of –NH_2_^+^CH_3_ in TAK-438-1) are shown in Figure 5 with the interaction modes at 0 ns and the time of the shortest distances (SCH28080: 7.570 ns, distance 2.744 Å; TAK-438-1: 3.180 ns, distance 6.218 Å). Due to the electrostatic repulsive interaction, the protonated form (+1 charged) can block K^+^ more easily than the neutral P-CABs. From 4.218 ns, the distance between K^+^ and TAK-438-1 is larger than 20 Å, while this time point is 9.421 ns for SCH28080 system (Figure 5A). Furthermore, analyses of root-mean-square fluctuation (RMSF) versus the residue number for SCH28080 and TAK-438-1complexes are illustrated in Figure 6. All complexes share similar RMSF distributions and similar trends of dynamic features. They are relatively rigid in the active site region (residues Leu143 in TM2, Ala337 in TM4, Tyr801 in TM5, Leu811 in the TM5-6 loop, and Cys815 in TM6) as reported in the literatures^18,33,47-50^ (residue IDs in pig H^+^,K^+^-ATPase correspondingly are Leu141, Ala335, Tyr799, Leu809, and Cys813). The fluctuation of the active site for TAK-438-1 complex (except for Leu143) is smaller than SCH28080 complex. RMSF values of the important amino acids in different P-CAB system are listed in Table 2.

**Table 2:** RMSF values (Å) of important amino acid residues in different P-CABs system.

	Leu143	Ala337	Tyr801	Leu811	Cys815
SCH28080	1.267	1.198	1.038	1.544	1.196
Soraprazan	1.205	1.138	0.998	1.280	1.243
Revaprazan	2.403	1.489	1.483	1.905	1.212
AZD0865	**1.075**	1.173	1.069	1.286	1.239
TAK-438	1.109	**0.985**	**0.837**	1.081	0.981
SCH28080-1	1.831	1.913	2.174	2.134	1.758
Soraprazan-1	1.637	1.260	1.005	1.193	1.340
Revaprazan-1	1.391	1.025	0.867	1.226	0.964
AZD0865-1	1.338	1.197	0.979	1.544	1.062
TAK-438-1	1.642	0.991	0.949	**1.067**	**0.865**

The smallest RMSF values of residues among P-CABs complexes are bold.

### Interaction modes of P-CABs with H^+^,K^+^-ATPase

**Figure 7:**
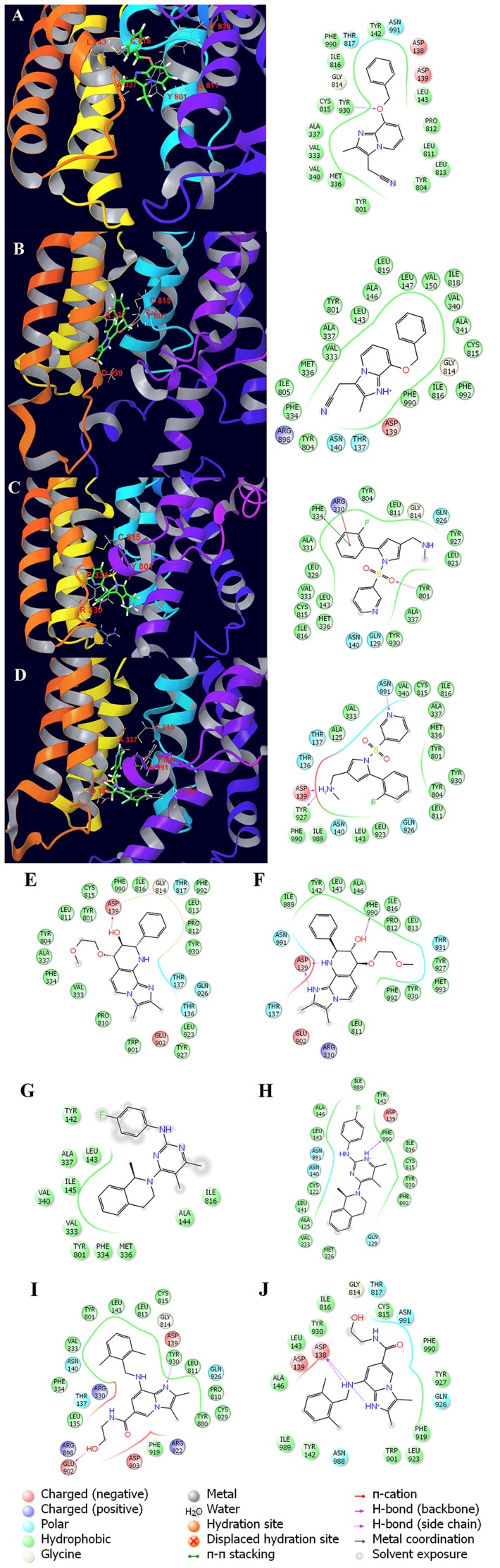
Interaction modes of P-CABs with H^+^,K^+^-ATPase. (A) SCH28080; (B) SCH28080-1; (C) TAK-438; (D) TAK-438-1; (E) Soraprazan; (F) Soraprazan-1; (G) Revaprazan; (H) Revaprazan-1; (I) AZD0865; (J) AZD0865-1

To investigate P-CAB interactions in the binding site, the average structures from last 1 ns MD trajectory were compared (Figure 7). After MD simulation, the pose of SCH28080 was very similar to that reported by Abe *et al*. [33]. The binding site is in the luminal cavity, surrounded by Ala337, Tyr801 and Cys815, which have hydrophobic interactions with SCH28080 (Figure 7A). The hydrogen bond (H-bond) interactions, which play an important role in P-CABs binding to H^+^,K^+^-ATPase, are listed in Table 3 and shown in Figure 7. Oxygen atom of SCH28080 and nitrogen atom of AZD0865 formed hydrogen bonds with Tyr930. TAK-438 was found to form H-bond with Tyr801, and has π- π stacking interaction with Phe334 and π-cation interaction with Arg330. The protonated forms have different interaction modes compared to the neutral P-CABs. There are strong H-bond interactions between Asp139 and TAK-438-1 (distance 1.727±0.012 Å) and Soraprazan-1 (distance 1.623±0.006 Å and 2.133±0.015 Å). Asp139 also has negative charged interactions with all protonated P-CABs (+1 charged). AZD0865-1 interacts with Asp138 via two H-bonds (distance 1.695±0.009 Å and 1.903±0.013 Å). Both Soraprazan-1 and Revaprazan-1 have H-bonds with Phe990. TAK-438-1 formed hydrogen bonds with Asp139, Tyr927 and Asn991 simultaneously.

**Table 3:** Hydrogen bond analysis after MD simulation.

P-CABs	Atoms	Residues	Atoms	Distance (Å)*
SCH28080	O1	Tyr930	HH	1.986±0.016
TAK-438	O2	Tyr801	HH	1.947±0.013
TAK-438-1	H16	Asp139	OD2	1.727±0.012
	H17	Tyr927	OH	2.188±0.019
	N2	Asn991	HD22	2.284±0.020
Soraprazan	H20	Asp139	OD2	1.767±0.009
Soraprazan-1	H12	Asp139	OD1	2.133±0.015
	H26	Asp139	OD1	1.623±0.006
	H20	Phe990	O	1.976±0.014
Revaprazan-1	H24	Phe990	O	1.898±0.012
AZD0865	H14	Glu902	OE2	1.931±0.025
	N2	Tyr930	HH	2.118±0.018
AZD0865-1	H15	Asp138	OD1	1.903±0.013
	H27	Asp138	OD1	1.695±0.009

*The average distance with standard error (SE = standard deviation/N^1/2^) of hydrogen bond in the last 1 ns MD.

### Binding free energy of P-CABs

**Figure 8:**
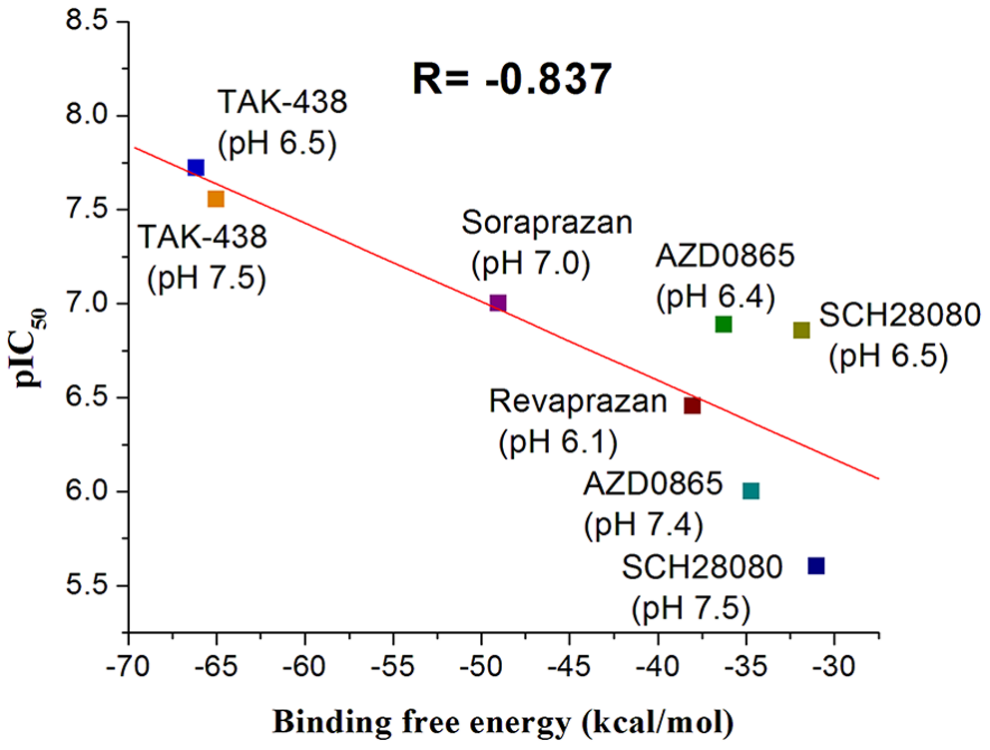
The relationship between binding free energy and pIC_50_ of P-CABs at different pH.

The binding free energies of all ten systems were calculated by MM/GBSA method. As listed in Table 4, the ΔG^'^_bind_ and ΔG_bind_ values all show that the binding affinity of protonated P-CABs is more favorable than that of neutral P-CABs. The order of favorable binding interaction is TAK-438-1>Soraprazan-1>Revaprazan-1>SCH28080-1>AZD0865-1>TAK-438>AZD0865>SCH28080≈Soraprazan>Revaprazan, which is consistent with the experimental results at different pH. According to the protonated form percentage of P-CABs in Table 1, the binding free energies (ΔG_bind_) of protonated and neutral mixtures of P-CABs at different pH values were calculated. The correlation coefficient R between ΔG_bind_ of the mixture and pIC_50_ (negative logarithms of 50% inhibition concentration) is −0.837 (Figure 8).

**Table 4:** The binding free energies of P-CAB complexes (kcal/mol).

P-CABs	ΔE_internal_	ΔE_Elect_	ΔE_VDW_	ΔG_GB_	ΔG_SA_	TΔS	ΔG^'^_bind_	ΔG_bind_
SCH28080	−0.00±0.00	−10.95±1.71	−33.74±3.64	16.86±1.64	−0.36±0.33	−0.88±0.13	−31.78±1.03	−30.90±1.01
SCH28080*	−0.00±0.00	−11.10±0.31	−41.48±0.43	23.43±0.52	−0.10±0.36	−1.17±0.25	−29.05±0.67	−27.88±0.74
SCH28080-1	−0.00±0.00	−77.53±1.74	−41.03±0.35	75.97±1.30	−2.26±0.21	−2.34±0.21	−44.86±0.89	−42.53±0.87
TAK-438	−0.00±0.00	−12.79±0.62	−40.04±0.40	15.64±0.33	−1.79±0.46	−2.95±0.25	−38.98±0.63	−36.03±0.59
TAK-438-1	−0.00±0.00	−145.88±1.80	−37.22±0.32	122.74±1.12	−9.61±0.30	−3.32±0.29	−69.97±1.36	−66.65±1.17
TAK-438-1*	−0.01±0.00	−145.48±3.34	−44.56±0.45	127.56±2.01	−7.82±0.50	−3.84±0.45	−70.31±1.91	−66.48±1.87
Soraprazan	−0.00±0.00	−17.98±0.56	−42.96±0.54	30.30±0.46	−1.79±0.38	−1.60±0.15	−32.43±0.68	−30.83±0.65
Soraprazan-1	−0.00±0.00	−100.27±2.38	−45.37±0.59	89.08±1.99	1.85±0.37	−2.98±0.21	−54.72±1.09	−51.74±1.04
Revaprazan	−0.00±0.00	−0.27±0.22	−25.84±0.52	8.81±0.30	−0.88±0.12	−2.13±0.25	−18.18±0.44	−16.05±0.42
Revaprazan-1	0.00±0.00	−89.72±1.61	−47.48±0.26	89.71±1.38	−2.88±0.29	−2.84±0.12	−50.36±0.81	−47.51±0.80
AZD0865	−0.00±0.00	−11.59±0.79	−50.47±0.47	24.86±0.59	−0.69±0.31	−3.45±0.53	−37.89±0.68	−34.44±0.94
AZD0865-1	−0.00±0.00	−111.31±2.13	−41.19±0.87	107.87±1.82	1.73±0.23	−2.93±0.27	−42.91±1.08	−39.98±0.96

*The binding free energies of P-CAB complexes after 100 ns disassociation molecular dynamics.

To estimate which energy term has most impact on the binding affinities, the four individual energy components (ΔE_Elect_, ΔE_VDW_, ΔG_GB_, and ΔG_SA_) were carefully compared. From Table 4, it can be seen that both the van der Waals (ΔE_VDW_) and the electrostatic (ΔE_Elect_) contributions are essential for P-CABs binding to H^+^,K^+^-ATPase. For the neutral form, the contributions of ΔE_VDW_ are more favorable than ΔE_Elect_ term. But for protonated P-CABs, the major favorable contributor is ΔE_Elect_ term, which is far larger than for the neutral form. Although polar solvation (ΔG_GB_) term of protonated form is also far larger than for the neutral form, which could offset ΔE_Elect_, the net electrostatic contributions (ΔE_Elect_+ΔG_GB_) are more favorable for the protonated compounds. Thus, the electrostatic contribution is more crucial for distinguishing the binding affinities among P-CAB complexes. Except for Soraprazan-1 and AZD0865-1, nonpolar solvation term (ΔG_SA_) contribute favorably to binding. Among all P-CABs, ΔE_Elect_ and ΔG_SA_ of TAK-438-1 is the most favorable (−145.88±1.80 and −9.61±0.30 kcal/mol). In addition, the contributions of the conformational entropy (TΔS) are very similar for the protonated compounds (−2.34±0.21∼−3.32±0.29 kcal/mol).

**Figure 9:**
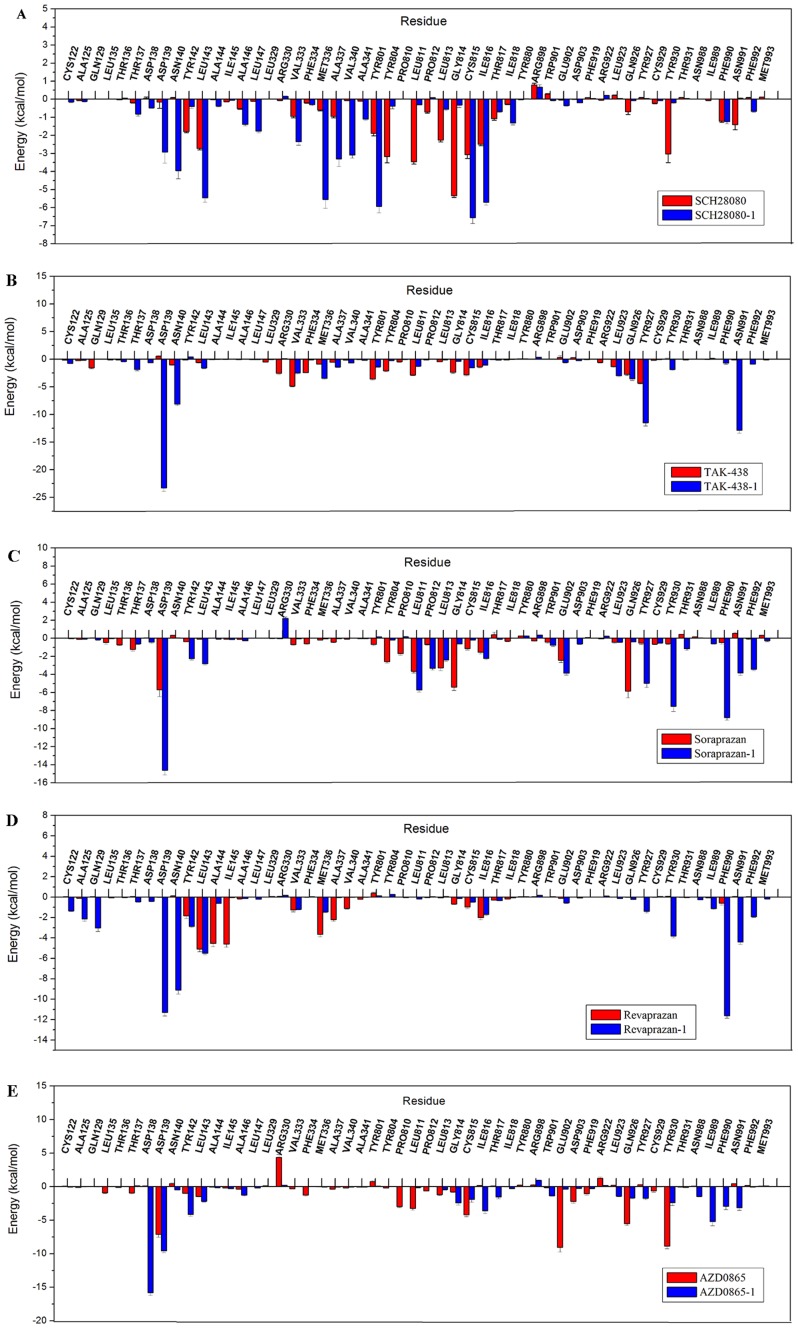
The comparison of energy decomposition for residues in binding sites of P-CABs. (A) SCH28080 and SCH28080-1; (B) TAK-438 and TAK-438-1; (C) Soraprazan and Soraprazan-1; (D) Revaprazan and Revaprazan-1; (E) AZD0865 and AZD0865-1

The binding free energy between the protein and P-CABs was decomposed into the contribution of each residue, which provides quantitative information of the key residues related to the detailed binding mechanism. From the energy comparison of residues in binding sites (Figure 9), it can be observed that there is the distinct difference between protonated and neutral forms of P-CABs. The energies of residues Tyr142, Tyr804, Leu811, Leu813, Gly814, and Tyr930 interacting with SCH28080-1 (−0.40±0.10, −0.39±0.15, −0.31±0.02, −0.55±0.07, −0.33±0.12, and −0.20±0.04 kcal/mol) are less favorable than those of SCH28080 complex (−1.79±0.07, −3.18±0.34, −3.46±0.13, −2.26±0.10, −5.35±0.09, and −3.02±0.49 kcal/mol). But the interactions between SCH28080-1 and residues Asp139, Asn140, Leu143, Val333, Met336, Ala337, Val340, Tyr801, Cys815, and Ile816 of H^+^,K^+^-ATPase (−2.92±0.62, −3.96±0.45, −5.46±0.25, −2.35±0.20, −5.55±0.48, −3.31±0.42, −3.09±0.17, −5.94±0.35, −6.56±0.31, and −5.70±0.15 kcal/mol) are more favorable than those of SCH28080 (−0.16±0.24, 0.08±0.03, −2.73±0.08, −0.95±0.08, −0.60±0.05, −0.95±0.08, −0.07±0.02, −1.89±0.13, −3.07±0.22, and −2.49±0.08 kcal/mol) (Figure 9A), which in particular include the key residues Leu143, Tyr801, and Cys815 (all above −5.00 kcal/mol for SCH28080-1). This might be the reason that the activity of SCH28080 at pH 6.5 is dramatically higher than that at pH 7.5. Due to strong hydrogen bond and electrostatic interactions, the energy contributions of Asp139 to protonated form are all more favorable than those to neutral P-CABs (−14.63±0.51 kcal/mol to Soraprazan-1, -11.29±0.35 kcal/mol to Revaprazan-1, −9.57±0.26 kcal/mol to AZD0865-1). In particular, the highest value (−23.28±0.64 kcal/mol) is reached for TAK-438-1. Even though less favorable than their protonated forms, the interactions with Asp139 are also strong to Soraprazan (−5.69±0.77 kcal/mol) by H-bond and to AZD0865 (−7.14±0.41 kcal/mol) by electrostatic interactions. Asp139 could be the key residue in the binding sites of P-CABs, which was not paid attention to previously. TAK-438 has more favorable interactions with the key residues Val333, Tyr801, Leu811, and Cys815 (−4.88±0.13, −3.59±0.16, −2.86±0.09, −2.84±0.17 kcal/mol). However, the binding free energy of TAK-438-1 is higher than TAK-438 because TAK-438-1 has strong interactions with Asp139 (H-bond and electrostatic interactions), Asn140 (polar interaction, −8.13±0.25 kcal/mol), Tyr927 (H-bond and hydrophobic interactions, −11.51±0.56 kcal/mol), and Asn991 (H-bond and polar interactions, −12.85±0.53 kcal/mol) (Figure 7D). Soraprazan-1 and Revaprazan-1 have almost the same features compared to TAK-438-1. Compared to their neutral form, they have more favorable interactions with residues Asp139, Asn140, Tyr142, Leu143, Tyr927, Tyr930, Phe990, Asn991, and Phe992. In addition, there is strong hydrophobic interaction between Soraprazan-1 and Leu811 (−5.72±0.24 kcal/mol). Different to the other P-CABs, Revaprazan-1 has hydrophobic interactions with Cys122 (−1.35±0.08 kcal/mol), Ala125 (−2.15±0.21 kcal/mol), and electrostatic interaction with Gln129 (−3.02±0.34 kcal/mol). As well as hydrophobic interactions with Pro810 (−2.97±0.13 kcal/mol), Leu811 (−3.26±0.24 kcal/mol), and Cys815 (−4.22±0.26 kcal/mol), AZD0865 interacts with Glu902 (−9.04±0.70 kcal/mol) and Tyr930 (−8.89±0.37 kcal/mol) by H-bonds, with Gln926 (−5.53±0.23 kcal/mol) by polar interaction, and with Asp139 by electrostatic interaction. Positively charged Arg330 has unfavorable interactions with AZD0865 and Soraprazan-1 (4.37±0.45 and 2.19±0.15 kcal/mol). Because of the strong H-bond and electrostatic interactions with nitrogen atoms, the energy contribution of Asp138 is high (−15.82±0.41 kcal/mol) for AZD0865-1, second only to the contribution of Asp139 for TAK-438-1. The results demonstrate that the hydrogen bond and electrostatic interactions with Asp139 (or Asp138) should be very important for P-CABs binding to H^+^,K^+^-ATPase, which are in agreement with the reference [62]. Using the competitive inhibitor 8-[(4-azidophenyl) methoxy]-1-trithiomethyl-2,3-dimethylimidazo-(1,2-a) pyrimidium iodide (mDAZIP, a photoactivable compound derived from SCH 28080), Munson *et al*. [62] suggested the binding site was on the luminal side between Gln129 and Asn140 (between Gln127 and Asn138 in the TM1-2 loop of pig H^+^,K^+^-ATPase).

### Disassociation molecular dynamics

**Figure 10:**
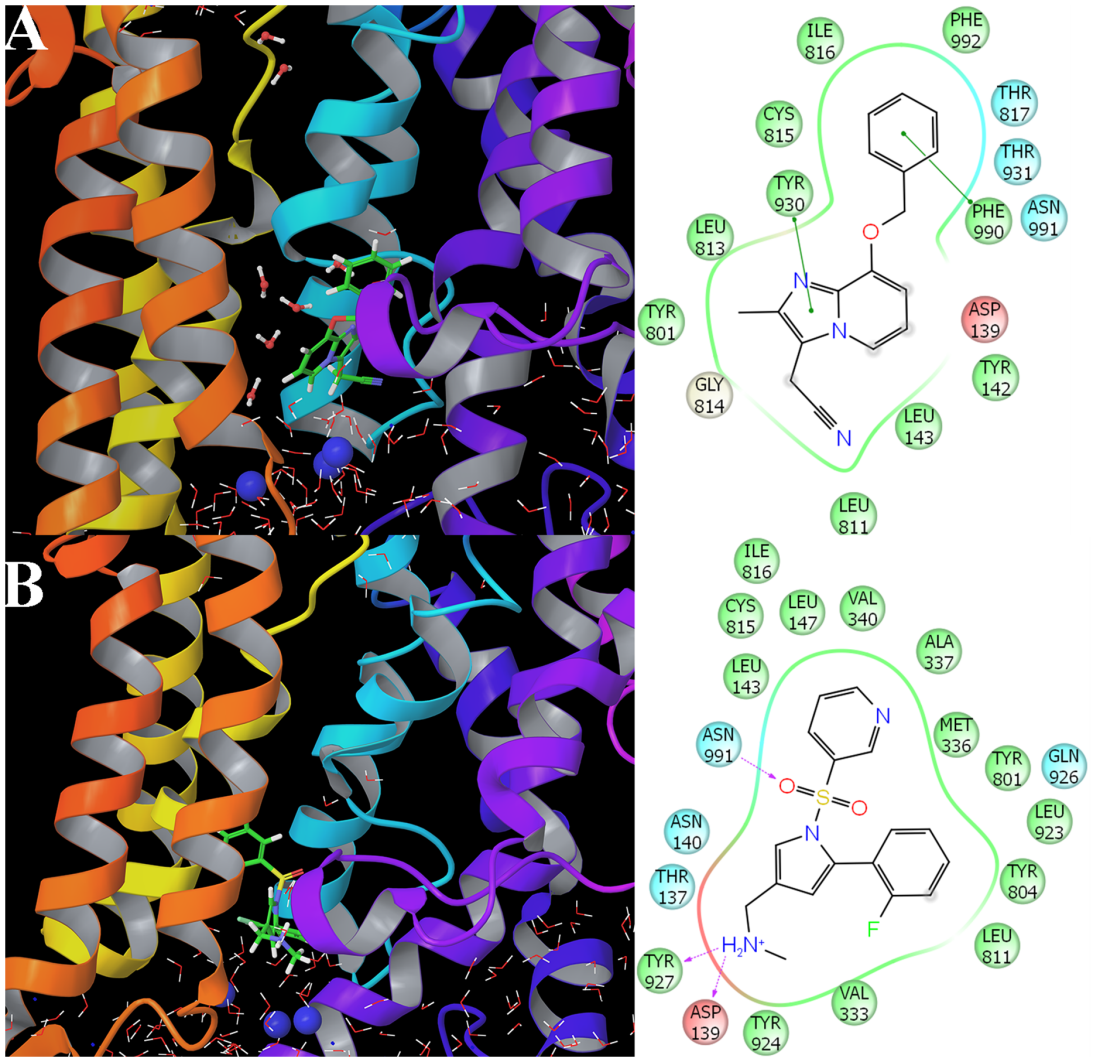
Interaction modes of SCH28080 (A) and TAK-438-1 (B) with H^+^,K^+^-ATPase after 100 ns disassociation molecular dynamics. Water molecules into the ion channel are shown in ball & stick model and the potassium ions near P-CABs are represented by blue ball.

**Figure 11:**
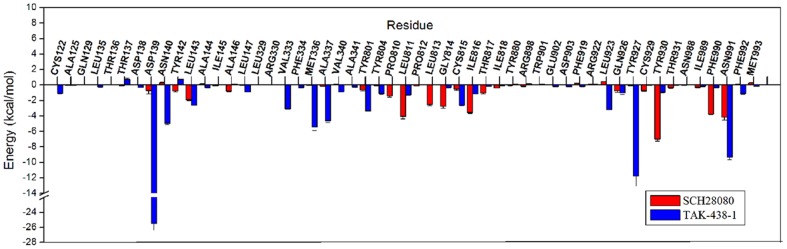
The comparison of energy decomposition for residues in binding sites of SCH28080 and TAK-438-1 after 100 ns disassociation molecular dynamics.

After 100 ns molecular dynamics (RMSD for the backbone atoms and RMSF for the residues of SCH28080 and TAK-438-1complexes are illustrated in Figure S2 and S3), SCH28080 occurred partial disassociation from H^+^,K^+^-ATPase and ΔG_bind_ of SCH28080 decreased to −27.88±0.74 kcal/mol (Table 4). Because water molecules have went into the ion channel, the interactions between SCH28080 and residues Val333 to Val340 in TM4 were cut off (Figure 10A and Figure 11). In contrast to SCH28080, ΔG_bind_ of TAK-438-1 after 100 ns MD was −66.48±1.87 kcal/mol (Table 4) and similar to that in 10 ns MD. TAK-438-1 still interacted with Asp139 (−25.48±0.90 kcal/mol), Tyr927 (−11.81±1.28 kcal/mol) and Asn991 (−9.33±0.31 kcal/mol) by H-bonds and blocked water molecules into the ion channel (Figure 10B and Figure 11). Therefore TAK-438-1 could have the long dwell time and disassociate from H^+^,K^+^-ATPase very slowly, which make it a likely competitor for PPIs.

## Conclusions

In the present study, to clarify the interaction mechanism between protonated P-CABs and H^+^,K^+^-ATPase, MD simulations and MM/GBSA binding free energy calculations were conducted. There is a distinct difference in interaction mode between protonated and neutral P-CABs. With positive charges on nitrogen atoms, protonated forms of P-CABs have a competitive advantage in blocking potassium ion into the luminal channel and binding to H^+^,K^+^-ATPase mostly via electrostatic interactions. According to the binding free energy analysis, the binding affinity of protonated forms is more favorable than that of neutral P-CABs. Asp139 in particular should be a very important binding site for protonated forms of P-CABs through hydrogen bonds and electrostatic interactions. The energy contributions of Asp139 for protonated forms are all more favorable than those for neutral P-CABs. Thus, protonated form is the potent form of P-CABs. To enhance P-CABs pKa values and binding with the key residue Asp139 would help increase the inhibition activity. The 100 ns disassociation molecular dynamics suggest that TAK-438 in protonated form could have the long dwell time and disassociate from H^+^,K^+^-ATPase very slowly, which make it a likely competitor for PPIs. The findings in this work provide a better structural understanding of the binding sites in H^+^,K^+^-ATPase and a basis for further rational design of novel P-CABs.

## Supporting Information

Figure S1 Ramachandran plot of the human H^+^,K^+^-ATPase model.
(TIF)

Figure S2 RMSD for the backbone atoms of the SCH28080 and TAK-438-1complexes in 100 ns disassociation molecular dynamics.
(TIF)

Figure S3 RMSF of each residue for SCH28080 and TAK-438-1 complexes in 100 ns disassociation molecular dynamics.
(TIF)

Table S1 Glide docking Gscore, Glide energy and QM/MM energy (kcal/mol) of P-CABs.
(DOC)
