# Supplementary figures and images for: Protonated Form: The Potent Form of Potassium-Competitive Acid Blockers
Source: PLoS One. 2014 May 20;9(5):e97688. doi: 10.1371/journal.pone.0097688 (PMC4028304; doi:10.1371/journal.pone.0097688)

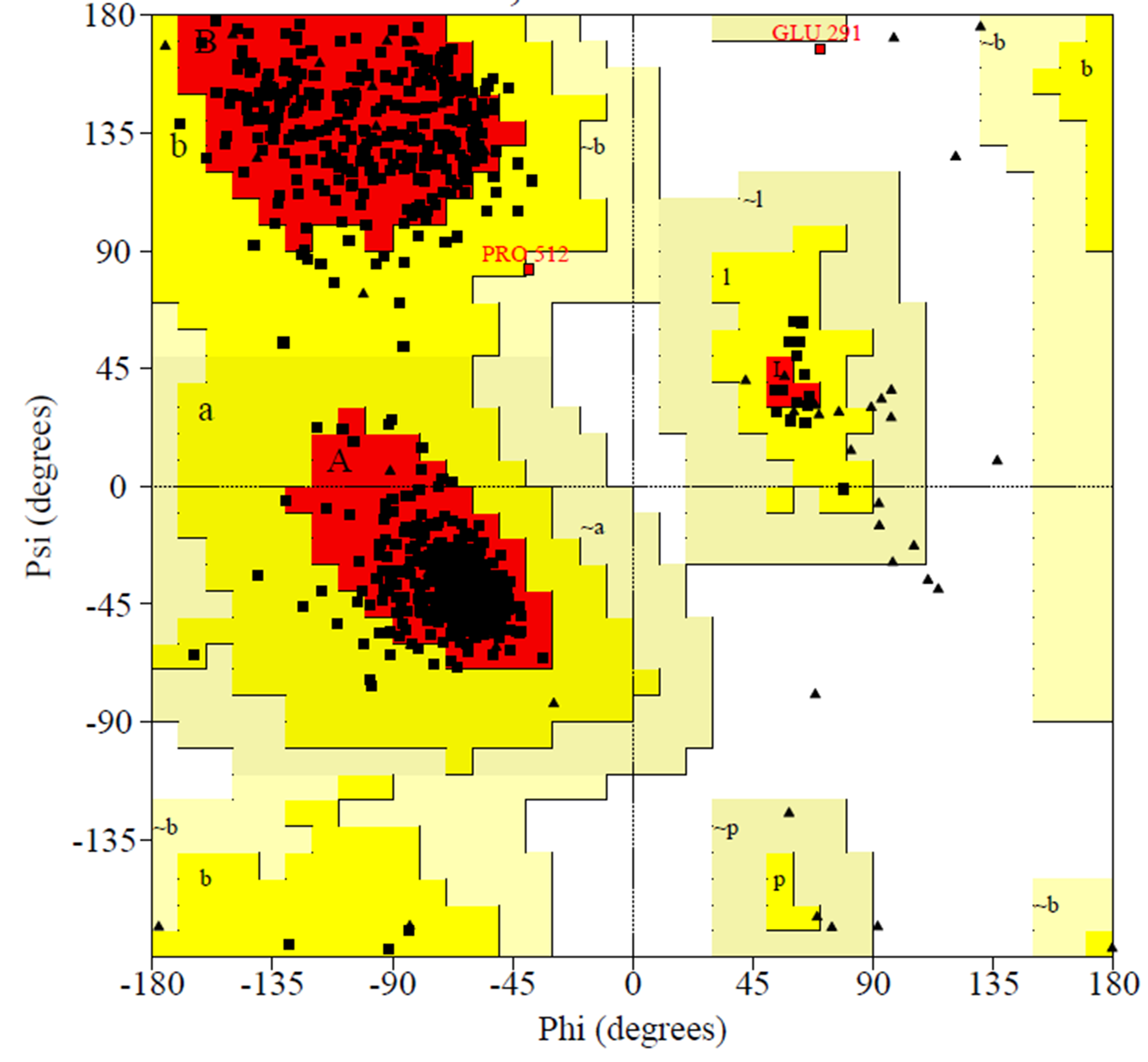

Supplement: Figure S1 — Ramachandran plot of the human H+,K+-ATPase model. (TIF) [file pone.0097688.s001.tif]

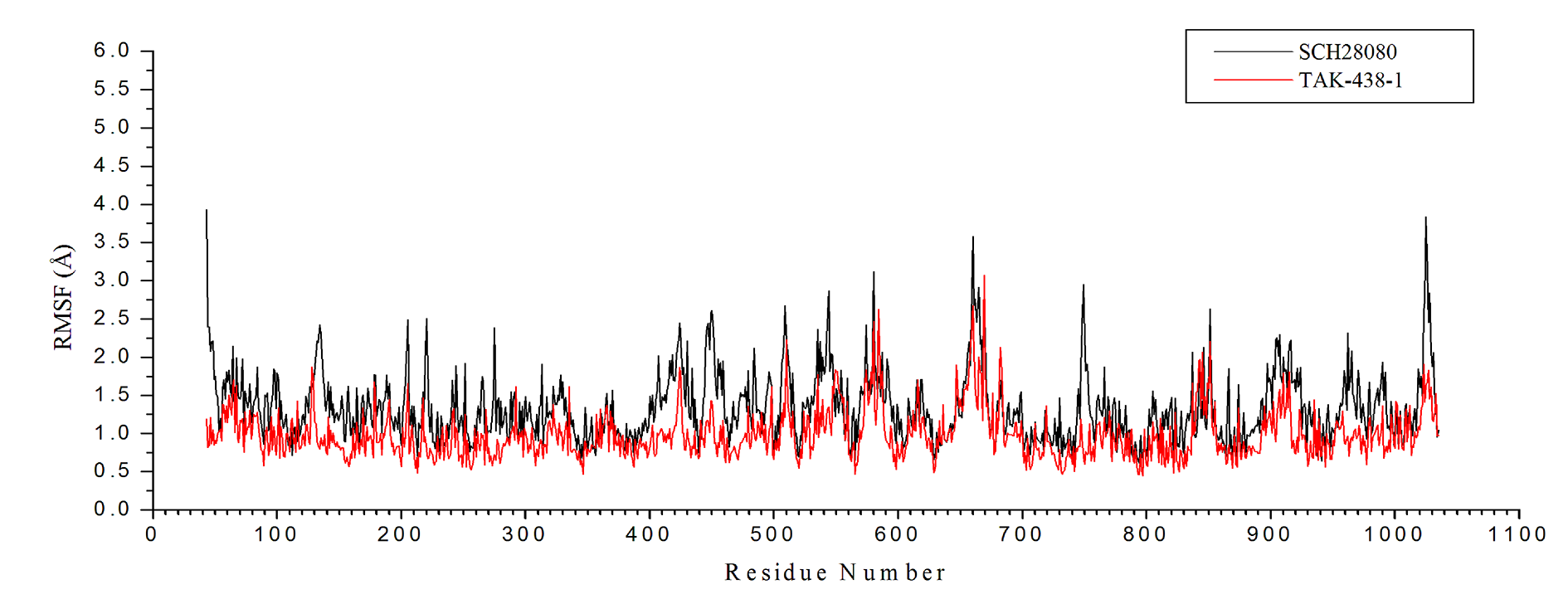

Supplement: Figure S3 — RMSF of each residue for SCH28080 and TAK-438-1 complexes in 100 ns disassociation molecular dynamics. (TIF) [file pone.0097688.s003.tif]
