# Supplementary material for: Protonated Form: The Potent Form of Potassium-Competitive Acid Blockers
Source: PLoS One. 2014 May 20;9(5):e97688. doi: 10.1371/journal.pone.0097688 (PMC4028304; doi:10.1371/journal.pone.0097688)
Supplement: Table S1 — Glide docking Gscore, Glide energy and QM/MM energy (kcal/mol) of P-CABs. (DOC) [file pone.0097688.s004.doc]

**Table S1**. Glide docking Gscore, Glide energy and QM/MM energy (kcal/mol) of P-CABs

| P-CABs | Gscore | Glide energy | QM/MM energy |
| --- | --- | --- | --- |
| SCH28080 | -5.33 | -34.17 | -966.41 |
| SCH28080-1 | -5.74 | -32.23 | -967.35 |
| TAK-438 | -5.36 | -36.15 | -1540.11 |
| TAK-438-1 | -6.29 | -39.56 | -1541.55 |
| Soraprazan | -6.32 | -40.89 | -1275.14 |
| Soraprazan-1 | -5.82 | -40.87 | -1275.82 |
| Revaprazan | -6.12 | -38.70 | -1241.03 |
| Revaprazan-1 | -5.55 | -38.81 | -1241.68 |
| AZD0865 | -6.41 | -35.03 | -1255.71 |
| AZD0865-1 | -5.97 | -45.89 | -1256.98 |
